# Microbial variations in sputum cultures among hospitalized patients with community-acquired pneumonia: differences in sputum microbiota between asthma and COPD patients

**DOI:** 10.36416/1806-3756/e20230329

**Published:** 2024-05-08

**Authors:** Fatih Uzer, Burcu Karaboğa, A.Gamze Çalış, Nermin Kaplan, Rojan Barış Gedik, Ahmet Alper Durmuş, Umut Barış Inanc, Metin Akgün

**Affiliations:** 1. Department of Chest Disease, Akdeniz University School of Medicine, Antalya, Turkey.; 2. Chest Disease Clinic, Ataturk State Hospital, Antalya, Turkey.; 3. Chest Disease Clinic, Antalya Training and Research Hospital, Antalya, Turkey.; 4. Department of Chest Disease, Agrı Ibrahim Cecen University, Agrı, Turkey.

**Keywords:** Community-acquired infections, Pulmonary disease, chronic obstructive, Asthma, Patient admission, Sputum, Culture techniques

## Abstract

**Objective:**

To assess differences in the sputum microbiota of community-acquired pneumonia (CAP) patients with either COPD or asthma, specifically focusing on a patient population in Turkey.

**Methods:**

This retrospective study included hospitalized patients > 18 years of age with a diagnosis of pneumonia between January of 2021 and January of 2023. Participants were recruited from two hospitals, and three patient groups were considered: CAP patients with asthma, CAP patients with COPD, and CAP patients without COPD or asthma.

**Results:**

A total of 246 patients with CAP were included in the study, 184 (74.8%) and 62 (25.2%) being males and females, with a mean age of 66 ± 14 years. Among the participants, 52.9% had COPD, 14.2% had asthma, and 32.9% had CAP but no COPD or asthma. Upon analysis of sputum cultures, positive sputum culture growth was observed in 52.9% of patients. The most commonly isolated microorganisms were Pseudomonas aeruginosa (n = 40), Acinetobacter baumannii (n = 20), Klebsiella pneumoniae (n = 16), and Moraxella catarrhalis (n = 8). CAP patients with COPD were more likely to have a positive sputum culture (p = 0.038), a history of antibiotic use within the past three months (p = 0.03), utilization of long-term home oxygen therapy (p < 0.001), and use of noninvasive ventilation (p = 0.001) when compared with the other patient groups. Additionally, CAP patients with COPD had a higher CURB-65 score when compared with CAP patients with asthma (p = 0.004).

**Conclusions:**

This study demonstrates that CAP patients with COPD tend to have more severe presentations, while CAP patients with asthma show varied microbial profiles, underscoring the need for patient-specific management strategies in CAP.

## INTRODUCTION

Pneumonia continues to be a significant public health concern, resulting in an annual mortality of over 3 million individuals.^1^ In the elderly, pneumonia constitutes 20-40% of hospital admissions, impacting an estimate of 2-13 individuals per 1,000 in the community. This results in heightened healthcare utilization, increased morbidity, and elevated mortality rates.^2^ Individuals with underlying obstructive lung diseases, such as asthma and COPD, are particularly susceptible to the development of pneumonia. Community-acquired pneumonia (CAP) encompasses a diverse range of microbiological agents as causative factors. *Streptococcus pneumoniae* is the most commonly identified pathogen, responsible for approximately 25% of cases.^3^ The causative agents of pneumonia may vary geographically.^4^ ^,^ ^5^ In a review compiling studies conducted in Asian countries, it was observed that pathogens such as *S. pneumoniae, Mycoplasma pneumoniae, Chlamydia pneumoniae, and Legionella pneumophila* are more prevalent in Western countries, whereas gram-negative bacilli and *Mycobacterium tuberculosis* are more commonly encountered in the Northeast.^4^

Chronic respiratory diseases such as COPD and asthma contribute to airway inflammation, airflow limitation, and increased sputum production, providing a favorable milieu for colonization and proliferation of microorganisms within the respiratory tract. Consequently, understanding the clinical manifestations and complications associated with pneumonia in patients with asthma and COPD is of utmost importance. Studies in the literature generally focus on sputum samples collected during exacerbations of both diseases.^6^ However, few investigations have suggested the possibility of divergent etiological agents in hospitalized CAP patients with either asthma or COPD.^7^ ^-^ ^10^ Additionally, microbiological investigation of CAP in the context of coexisting asthma and COPD has not been sufficiently explored in Turkey.

A review by Beasley et al.^6^ highlighted that the bacterial flora in COPD patients varies with the severity of the condition. During stable periods and exacerbations in severe COPD, gram-negative organisms such as *Pseudomonas aeruginosa* are more common. Meanwhile, the presence of *Haemophilus parainfluenzae* and *Staphylococcus aureus* is less frequent, and their significance in COPD remains debatable.^6^ In contrast, studies focusing specifically on COPD patients with CAP have revealed a different microbial pattern. In the study by Pascual-Guardia et al.,^11^ *P. aeruginosa* was identified as the most frequently isolated microorganism in patients with bronchiectasis, low FEV_1_ levels, and recent hospitalization. However, Sethi reported that *Haemophilus influenzae* was the most common microorganism causing exacerbations in COPD patients.^12^ Separately, a study by Bari et al.^7^ explored the microbial landscape in COPD exacerbations. They found culture positivity in 65% of such patients, with *P. aeruginosa* again being the most frequently isolated microorganism.^7^ This distinction between the microbial profiles of COPD exacerbations and CAP patients with COPD underscores the complexity of microbial involvement in COPD. In the realm of asthma, in 27% of asthma patients with worsened symptoms, organisms such as *S. pneumoniae, Streptococcus pyogenes, S. aureus, Moraxella catarrhalis*, and *H. influenzae* were identified.^13^ The use of 16S rRNA sequencing has further elucidated bacterial taxa associated with different inflammatory phenotypes in asthma, such as dominance of *Streptococcus* spp. in eosinophilic asthma and of *H. influenzae* in neutrophilic asthma.^14^ ^,^ ^15^ However, the microbial profile in CAP patients with asthma does not show significant differences in etiological pathogens,^16^ suggesting a complex interplay between these respiratory conditions and their associated microbial environments.

The primary aim of this study was to assess differences in the sputum microbiota of CAP patients with either COPD or asthma, specifically focusing on a patient population in Turkey.

## METHODS

This retrospective study included hospitalized patients > 18 years of age diagnosed with CAP between January of 2021 and January of 2023. Participants were drawn from the Chest Disease clinics at the Akdeniz University School of Medicine and the Antalya Atatürk State Hospital. The study comprised three distinct patient groups: CAP patients with asthma, CAP patients with COPD, and CAP patients without either COPD or asthma. Inclusion criteria encompassed patients diagnosed with COPD following the GOLD guidelines.^17^ Within the asthma group, individuals exhibiting variable symptoms, such as wheezing, shortness of breath, cough, chest tightness, and variable expiratory airflow limitation, and who had previously received a formal diagnosis of asthma and undergone treatment for the disease were included in the study.^18^

**Figure 1:**
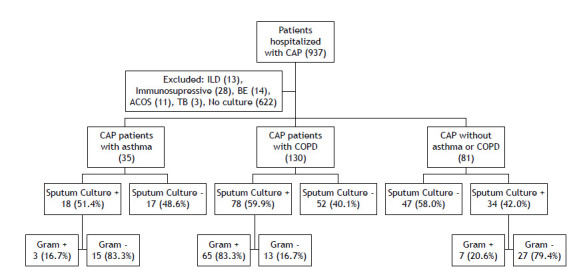
Flow chart of the patients. CAP: community-acquired pneumonia; ILD: interstitial lung disease; BE: bronchiectasis; ACOS: asthma-COPD overlap syndrome; and TB: tuberculosis.

The data of 937 patients who had been hospitalized for the treatment of CAP were evaluated. Patients who were immunosuppressed, had interstitial lung disease, were receiving treatment for active tuberculosis, had asthma-COPD overlap syndrome, were diagnosed with bronchiectasis, and those for whom cultures were not obtained were excluded, resulting in a total of 246 patients included in the study. The flow chart of the study is presented in Figure 1.

The data of the patients were retrospectively evaluated by accessing their hospital records and electronic medical records. Age, gender, smoking history, use of inhaled corticosteroids prior to hospital admission, comorbidities, sputum culture results, radiological data, and 30-day mortality rates were recorded for all patients. Sputum cultures were obtained prior to initiating antibiotic treatment. Sputum cultures reached the laboratory within 30 minutes in both centers and were examined by experienced technicians. Inadequate samples were not processed by the laboratories and were not reported in the database. No data were available on viral pathogens.

To be classified as pneumonia cases, patients needed to meet the following criteria within the first 48 h of hospital admission: identification of pulmonary infiltrations on chest X-ray or CT, presence of productive or dry cough, body temperature above 37.8°C or hypothermia below 36°C, and presence of at least one systemic inflammatory marker (leukocytosis > 10,000 mm^3^, leukopenia < 4,000 mm^3^, and elevated CRP or procalcitonin values).^11^

We used the mental Confusion, Urea, Respiratory rate, Blood pressure, and age = 65 years (CURB-65) scoring system to assess the risk of mortality in CAP patients. A score of 1 is given to each of the criteria: presence of mental confusion; blood urea nitrogen level > 7 mmol/L or > 20 mg/dL; RR > 30 breaths/min; systolic blood pressure < 90 mmHg or diastolic blood pressure < 60 mmHg; and age > 65 years.^19^ Scores were categorized as follows: a score of 0 to 1 indicates low risk, the patient being typically managed as an outpatient; a score of 2 is considered intermediate risk, suggesting the need for close monitoring or a short hospital stay; and a score of 3 to 5 is classified as high risk, generally requiring hospitalization and possible intensive care.

The study received ethical approval from the Non-interventional Ethics Committee of the Faculty of Medicine, Akdeniz University, on April 5, 2023 (decision number 293).

### Statistical analysis

Statistical analysis of the data was conducted using the IBM SPSS Statistics software package, version 23.0 (IBM Corporation, Armonk, NY, USA). Categorical variables were described as absolute and relative frequencies, while continuous variables were described as means and standard deviations. The normality of the data distribution was assessed using the Kolmogorov-Smirnov test. For normally distributed data, the means of two groups were compared using the Student’s t-test, and the means of more than two groups were compared using one-way ANOVA. For non-normally distributed data, the medians of two groups were compared using the Mann-Whitney U test, and the significance of categorical variables was analyzed using the chi-square test. The correlation between continuous variables was evaluated using the Spearman’s correlation test. A significance level of 0.05 was considered for statistical significance in the study.

## RESULTS

The study involved a total of 246 patients, comprising 184 males (74.8%) and 62 females (25.2%), with a mean age of 66.0 ± 14.3 years. Among them, 130 (52.9%) had COPD, 35 (14.2%) had asthma, and 81 (32.9%) had CAP without either COPD or asthma. Additionally, 150 (60.6%) of the patients had a history of smoking, and 120 (48.8%) had a history of hospitalization in the previous year. The proportion of males was higher in CAP patients with COPD when compared with CAP patients with asthma and CAP patients with no asthma or COPD (p < 0.001), as was the history of smoking more prevalent in that group (p < 0.001). Furthermore, CAP patients with COPD had a significantly higher rate of antibiotic use within the past three months (p = 0.01), a higher prevalence of long-term home oxygen therapy (p < 0.001), and an increased use of noninvasive ventilation (NIV) when compared with the other groups (p = 0.001). Before the onset of pneumonia, 30 (85.7%) of patients with asthma and 110 (84.6%) of patients with COPD were utilizing inhaled corticosteroids. Demographic and clinical characteristics of patients are presented in Table 1, and the outcomes of the study are presented in Table 2.

**Table 1:** Demographic and clinical characteristics of patients.^a^

Characteristic	CAP patients with asthma	CAP patients with COPD	CAP patients with no asthma or COPD	Total sample	p
	(n = 35)	(n = 130)	(n = 81)	(N = 246)	
Gender, male^†^	14 (40.0)	117 (90.0)	53 (65.4)	184 (74.8)	< 0.001*
Age, years	62.1 ± 16.5	68.0 ± 10.8	65.2 ± 17.4	66.2 ± 14.3	0.090
Current or former smoker^†^	9 (25.7)	105 (80.8)	35 (43.2)	149 (60.6)	< 0.001*
Coronary artery disease^†^	27 (77.1)	87 (66.9)	61 (75.3)	71 (28.9)	0.297
Hypertension^†^	12 (34.3)	68 (52.3)	39 (48.1)	127 (51.6)	0.166
Diabetes mellitus^†^	24 (68.6)	94 (72.3)	54 (66.7)	74 (30.1)	0.674
Hospitalization within the previous year^†^	17 (48.6)	71 (54.6)	32 (39.5)	120 (48.8)	0.102
Antibiotic use (last 3 months) ^a^	11 (31.4)	65 (50.0)	22 (27.2)	98 (39.6)	0.010*
ICS use^†^	30 (85.7)	110 (84.6)	-	139 (56.5)	0.872
LTOT^†^	5 (14.3)	38 (29.2)	3 (3.7)	46 (18.7)	< 0.001*
Homebound intensive care patient^†^	1 (2.9)	8 (6.2)	13 (16.0)	22 (8.9)	0.069
NIV^†^	14 (40.0)	77 (59.2)	27 (33.3)	118 (48.0)	0.001*
CURB-65 score^‡^	1.1 ± 0.7	1.5 ± 0.9	1.2 ± 1.0	1.3 ± 0.94	0.029*

CAP: community-acquired pneumonia; ICS: Inhaled corticosteroid, LTOT: long-term oxygen therapy; NIV: noninvasive ventilation, CURB-65: mental Confusion, Urea, Respiratory rate, Blood pressure, age > 65 years. ^a^Data are expressed as n (%) or as mean ± SD. *They were statistically significantly higher in COPD patients with CAP when compared with the other groups. The data conforms to a normal distribution as confirmed by the Kolmogorov-Smirnov test. ^†^Chi-square test. ^‡^Kruskal-Wallis test.

**Table 2:** Study outcomes.^a^

Outcome	CAP patients with asthma	CAP patients with COPD	CAP patients with no asthma or COPD	p
	(n = 35)	(n = 130)	(n = 81)	
Culture positivity^†^	17 (51.4)	78 (59.9)	34 (42.0)	0.038*
Gram staining, gram-positive^†^	3 (8.6)	15 (11.5)	7 (8.6)	0.156
Length of hospitalization, days^‡^	10.3 ± 8.1	11.0 ± 10.8	15.1 ± 15.4	0.078
Mortality rate^†^	0 (0.0)	11 (8.5)	4 (4.9)	0.155

a Data were expressed as n (%) or as mean ± SD. *Statistically significant increase in CAP patients with COPD. ^†^Chi-square test. ^‡^Kruskal-Wallis test.

**Figure 2:**
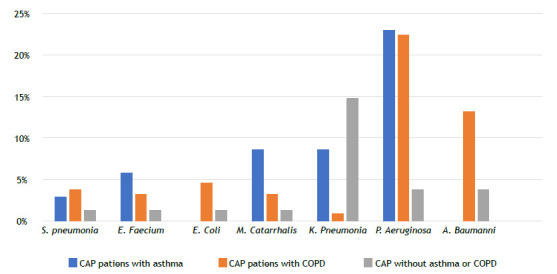
Sputum culture growths in patients hospitalized with community-acquired pneumonia (CAP).

**Figure 3:**
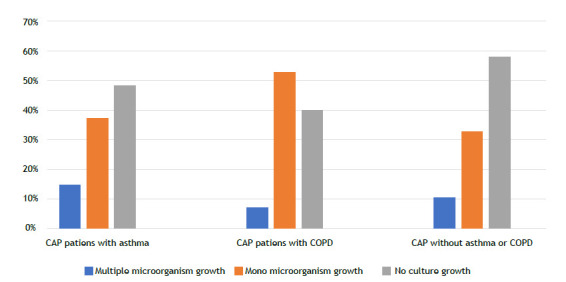
Multiple growth in sputum cultures of patients. The chi-square test showed that community-acquired pneumonia (CAP) patients with asthma had a significantly higher rate of multiple organisms (p = 0.031).

**Figure 4:**
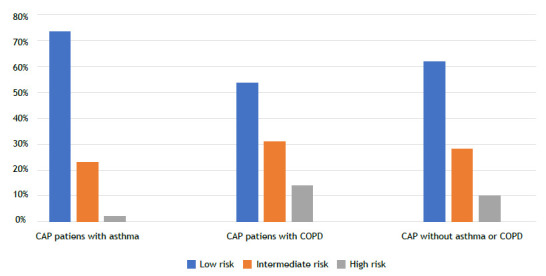
Distribution of CURB-65 scores among community-acquired pneumonia (CAP) patients, highlighting higher scores in CAP patients with COPD when compared with CAP patients with asthma (p = 0.004; Kruskal-Wallis test. The CURB-65 score categorizes patients into low- (0-1 points), intermediate- (2 points), and high- (3-5 points) risk groups.

Among the patients hospitalized with CAP, 622 (66.4%) had no sputum cultures performed, while among those who had sputum cultures obtained (n = 246), 130 samples (52.9%) showed growth. Sputum cultures were positive in 18 (51.4%) of CAP patients with asthma; in 78 (59.9%) of CAP patients with COPD; and in 34 (42.0%) of CAP patients with no asthma or COPD (p = 0.038; Figure 1). Among the positive sputum cultures, the most commonly isolated microorganisms were *P. aeruginosa* (n = 40), *Acinetobacter baumannii* (n = 20), *Klebsiella pneumoniae* (n =16), and *M. catarrhalis* (n = 8). The distribution of microorganism growth according to the underlying disease is shown in Figure 2. In CAP patients with asthma, the most frequently isolated microorganisms were *P. aeruginosa* (n = 8; 22.9%), *K. pneumoniae* (n = 3; 8.6%), and *M. catarrhalis* (n = 3; 8.6%). In CAP patients with COPD, the most commonly isolated microorganisms in cultures were *P. aeruginosa* (n = 29; 22.3%), *A. baumannii* (n = 17; 13.1%), *Escherichia coli* (n = 6; 4.6%), and *S. pneumoniae* (n = 5; 3.8%). However, in CAP patients with no asthma or COPD, the most commonly isolated microorganisms were *K. pneumoniae* (n = 12; 14.8%), *P. aeruginosa* (n = 3; 3.7%), and *A. baumannii* (n = 3; 3.7%). CAP patients with asthma had a significantly higher rate of multiple organism growth in sputum (14.3%) when compared with CAP patients with COPD (6.9%) and CAP patients with no asthma or COPD (9.9%; p = 0.031; Figure 3). The mean CURB-65 score of the patients included in the study was found to be 1.30 ± 0.94, and it was higher in patients with COPD when compared with patients with asthma (p = 0.004; Figure 4). Patients with positive sputum culture showed a higher tendency to be homebound intensive care patients (p = 0.040), receive long-term oxygen therapy (p < 0.001), and have a higher CURB-65 score (p = 0.014). Patients with a medical history of diabetes mellitus (p = 0.017) and hypertension (p = 0.026) had a lower incidence of positive sputum culture. When patients with multiple microorganism growth in sputum cultures were compared with those having a single microorganism growth, it was observed that the smoking history was significantly higher in the group with a single microorganism growth (35 pack-years vs. 20 pack-years; p = 0.016). Apart from this, no significant differences were found in the demographic and clinical characteristics between patients with multiple microorganism growth and those with a single microorganism growth.

## DISCUSSION

This study showed that CAP patients with COPD exhibited a higher predominance of males and smokers, along with increased antibiotic use and reliance on long-term home oxygen therapy and NIV. Sputum cultures revealed higher culture positivity in CAP patients with COPD. The most common microorganisms in sputum culture were identified as *P. aeruginosa, A. baumannii, K. pneumoniae*, and *M. catarrhalis*. The frequency of microorganism growth varied according to the underlying disease. In CAP patients with COPD, the most identified microorganisms were *P. aeruginosa* and *A. baumannii*. In CAP patients with asthma, the most frequently identified microorganisms were *P. aeruginosa, K. pneumoniae*, and *M. catarrhalis*. Patients with a positive sputum culture were more likely to require homebound intensive care and long-term oxygen therapy, and exhibited higher CURB-65 scores, whereas those with a history of diabetes mellitus and hypertension demonstrated a lower incidence of positive sputum cultures. Smoking was more pronounced in patients with a single microorganism growth in sputum cultures.

COPD affects more than 250 million people and is a leading cause of death for millions worldwide.^11^ ^,^ ^17^ CAP in COPD patients is associated with increased mortality and imposes a significant burden on hospitalizations and healthcare costs. Pneumonia treatment guidelines recommend empirical antibiotic treatment targeting *P. aeruginosa* in patients with underlying chronic lung diseases.^20^ In fact, a recent multicenter international study by Pascual-Guardia et al.^11^ reported *P. aeruginosa* as the most frequently isolated microorganism in CAP patients with COPD. Similarly, in our study, *P. aeruginosa* was the most commonly isolated microorganism in both CAP with asthma patients and CAP with COPD patients.

Patients with CAP may be infected by multiple microorganisms. In a study by Wark et al.,^21^ multiple microorganism growth was detected in 4 (9.8%) out of 45 patients with obstructive lung disease. The same study also found that COPD patients with signs of infection had a longer hospital stay when compared with asthma patients. A review by Yu et al.^22^ highlighted that COPD patients hospitalized with CAP tend to have a higher incidence of ICU admissions, increased need for mechanical ventilation support, and a higher mortality rate. In our study, it was observed that asthma patients had a higher frequency of multiple organism growth in sputum cultures than did COPD patients. Furthermore, CAP patients with COPD had a higher CURB-65 score than did the other groups. This suggests that the severity of pneumonia-related illness may be higher in COPD patients. In COPD patients, there is a higher likelihood of being male, having a smoking history, having recent antibiotic use, being on long-term oxygen therapy, using home NIV, and having an increased probability of microbial growth in sputum cultures when compared with asthma patients. These findings suggest that CAP patients with COPD may require different approaches in treatment.

The coexistence of pneumonia and COPD has been found to be associated with increased mortality.^22^ ^,^ ^23^ In a case-control study conducted in Switzerland, the coexistence of asthma or COPD with pneumonia has resulted in lower mortality rates when compared with a control group, contrary to the existing literature.^24^ There was no statistically significant different difference in mortality among the three groups of patients in our study 

*A. baumannii* and *E. coli* showed a predominance in sputum cultures of CAP patients with COPD, whereas *M. catarrhalis* and *Enterococcus faecium* were more common in those with asthma. Additionally, there was no significant difference in the prevalence of *P. aeruginosa* between CAP patients with COPD or with asthma. Furthermore, *K. pneumoniae* was found to be more prevalent in sputum cultures of CAP patients with no asthma or COPD. Our study results indicate differences in sputum culture growth between CAP patients with asthma and with COPD, a higher frequency of multiple organism growth being observed in CAP patients with asthma. These findings highlight the necessity for reevaluation of treatment strategies and approaches in this at-risk patient group for pneumonia.

According to guidelines, routine sputum culture is not recommended for all patients hospitalized with pneumonia. The decision to obtain a sputum culture should be based on various factors, such as the severity of illness, presence of risk factors, and clinical judgment of the healthcare provider.^20^ In an assessment of the diagnostic utility of sputum culture in CAP, only 15.8% of a total of 1,669 patients included in the study received a microbiological diagnosis.^25^ However, more than 40% of the patients did not undergo sputum specimen collection, and, of the specimens collected, 46% were deemed inadequate and were therefore not subjected to culture analysis.^25^ In our study, sputum culture was not performed in 66.4% of the patients. Among the patients who had sputum cultures performed, growth was detected in 52.9% of them. Since our primary aim was to assess culture growth, the evaluation of treatment modifications based on culture results was not conducted. Therefore, it is not possible to make a definitive interpretation regarding whether cultures are necessary for all patients hospitalized with CAP.

Although our research was conducted in only two centers and with a limited number of cases, it offers valuable insights into the etiology of CAP in Turkey. This approach will facilitate a careful and measured interpretation of our findings. We did not assess the impact of microbiological culture results on treatment decisions and on the evaluation of airflow limitation in obstructive lung diseases, constituting additional constraints. Nevertheless, the study elucidated the distinct course of pneumonia among asthma and COPD patients, highlighting the significance of the microbiological profile in shaping treatment and management strategies. To strengthen future research further, we recommend larger sample sizes, microbiota analysis, and diverse clinical parameters for more comprehensive assessments. Additionally, exploring treatment outcomes, disease severity, and long-term prognosis differences among patient groups will yield valuable insights.

In conclusion, this study reveals distinct patterns in sputum culture growths in CAP patients with asthma or with COPD. Specifically, CAP patients with COPD tend to have higher CURB-65 scores, indicating a potentially more severe disease course, while those with asthma often show multiple culture growths. These findings suggest a link between specific microorganisms and the clinical manifestations and complications of CAP in these patient groups. Consequently, for CAP patients with COPD, a heightened awareness of severity is recommended, and for those with asthma, treatment strategies should consider the likelihood of multiple organisms.
